# The role of blood metabolites in oral cancer: insights from a Mendelian randomization approach

**DOI:** 10.3389/fonc.2024.1305684

**Published:** 2024-02-05

**Authors:** Ziyang Hu, Zhe Xu, Qu Yue, Xuhong Pan, Ping Shi, Dandan Zhang, Jiexia Zhang, Runzhi Deng, Zitong Lin

**Affiliations:** ^1^ Shenzhen Longhua District Central Hospital, Department of Stomatology, Shenzhen, China; ^2^ Department of Oral and Maxillofacial Surgery, Nanjing Stomatological Hospital, Medical School of Nanjing University, Nanjing, China; ^3^ Department of Dentomaxillofacial Radiology, Nanjing Stomatological Hospital, Medical School of Nanjing University, Nanjing, China

**Keywords:** Mendelian randomization, blood metabolites, oral cancer, genome-wide association study, SNPs, metabolomics

## Abstract

**Aim:**

This research aimed to explore the causal impact of blood metabolites on oral cancer using a two-sample Mendelian randomization (MR) analysis. The study endeavored to identify potential biomarkers for oral cancer’s clinical management.

**Materials and methods:**

Based on the large individual-level datasets from UK Biobank as well as GWAS summary datasets, we first constructed genetic risk scores (GRSs) of 486 human blood metabolites and evaluated the effect on oral cancer. Various statistical methods, including inverse variance weighted (IVW), MR-Egger, and weighted median, among others, were employed to analyze the potential causal relationship between blood metabolites and oral cancer. The sensitivity analyses were conducted using Cochran’s Q tests, funnel plots, leave-one-out analyses, and MR-Egger intercept tests.

**Results:**

29 metabolites met the stringent selection criteria. Out of these, 14 metabolites demonstrated a positive association with oral cancer risk, while 15 metabolites indicated a protective effect against oral cancer. The IVW-derived estimates were significant, and the results were consistent across different statistical methodologies. Both the Cochran Q test and the MR-Egger intercept test indicated no heterogeneity and pleiotropy.

**Conclusion:**

This MR study offers evidence of the role specific blood metabolites play in oral cancer, pinpointing several with potential risk or protective effects. These findings could be helpful for new diagnostic tools and treatments for oral cancer. While the results are promising, additional research is necessary to fully validate and refine these conclusions. This study serves as a foundational step towards more comprehensive understandings in the future.

## 1 Introduction

Oral cancer, classified under head and neck malignancies, stands as the world’s sixth most common cancer, with oral squamous cell carcinoma constituting over 90% of these cases (1, 2). Several oral lesions, such as lichen planus, leukoplakia, erythroplakia, and oral sub-mucous fibrosis, have been recognized as potential precursors to malignant oral disorders (3). With limited understanding of oral cancer’s pathophysiology, emphasizing early detection is crucial to enhance survival rates.

Recently, the merger of metabolomics into systems biology has provided a novel approach to understanding disease processes. Through metabolomics, it’s possible to discern the biological complexity of diseases by identifying changes in metabolites and metabolic routes. Metabolic reprogramming is a distinctive feature of cancer cells, as many studies have highlighted (4–7). Interestingly, these cells prefer glycolysis for their energy needs, even in oxygen-rich conditions, a shift known as the Warburg effect or aerobic glycolysis (5). This metabolic shift contributes to both the initiation and progression of cancer. Besides the Warburg effect, cancer cells undergo diverse metabolic transformations, including shifts in lipid and amino acid pathways, such as glutaminolysis (4, 6, 7). Nonetheless, a thorough understanding of oral cancer’s metabolic traits remains elusive. Investigating these metabolic changes linked to oral cancer could lead to the discovery of new biomarkers and a deeper comprehension of the disease’s development and advancement. Recent advancements have combined metabolomics with extensive genotyping, enabling the examination of the relationship between genetic variations and metabolic phenotypes through genome-wide association studies (GWASs) (8, 9). Numerous genetic locations connected to metabolic phenotypes have been identified through these studies (8). By utilizing extensive GWAS data, genetic risk scores (GRSs) and Mendelian randomization (MR) have become instrumental in unraveling the etiology of complex diseases by effectively managing unidentified variables (10).

MR, an emerging analytical tool, uses genetic variations associated with exposures to assess the potential causal effects of these exposures on observed outcomes (11). Notably, MR is often likened to a “natural” randomized controlled trial (RCT) because alleles are randomly distributed during gamete production, possibly minimizing confounders and biases due to reverse causality (12). The enduring nature of genetic influences, together with the extensive GWAS data on oral diseases (13–19), makes MR studies an effective alternative to conventional, long-term clinical trials focused on preventing oral cancer, avoiding their significant costs and lengthy monitoring periods.

MR design has been applied to identify the potential mediators of oral cancer and related cancer. Gormley et al.’s study showed strong evidence of smoking’s causal effect on oral/oropharyngeal cancer and suggested an underestimated effect of alcohol when adjusted for smoking (14). Also, Chen et al. identified a possible causal link between inflammatory bowel disease and oral cavity cancer (15). For the relationship between cancers and blood metabolomic, Guo et al. applied two-sample MR to identify blood metabolites associated with lacunar stroke, finding 15 known and 14 unknown metabolites with potential implications in disease pathogenesis (20). Liu et al. utilized bidirectional Mendelian randomization on Chinese individuals, revealing 58 causal relationships between the gut microbiome and blood metabolites, with a notable link between fecal Oscillibacter and Alistipes and decreased triglyceride concentration (21).

However, to our knowledge, there is no MR study to assess the causal role of blood metabolites on oral cancer. In this study, we performed the first two-sample MR analysis of the GWAS summary data containing blood metabolites and oral cancer, revealed the causal impact of blood metabolites on oral cancer, provided new biomarkers for the clinical management of oral cancer.

## 2 Materials and methods

### 2.1 Study design

**Figure 1:**
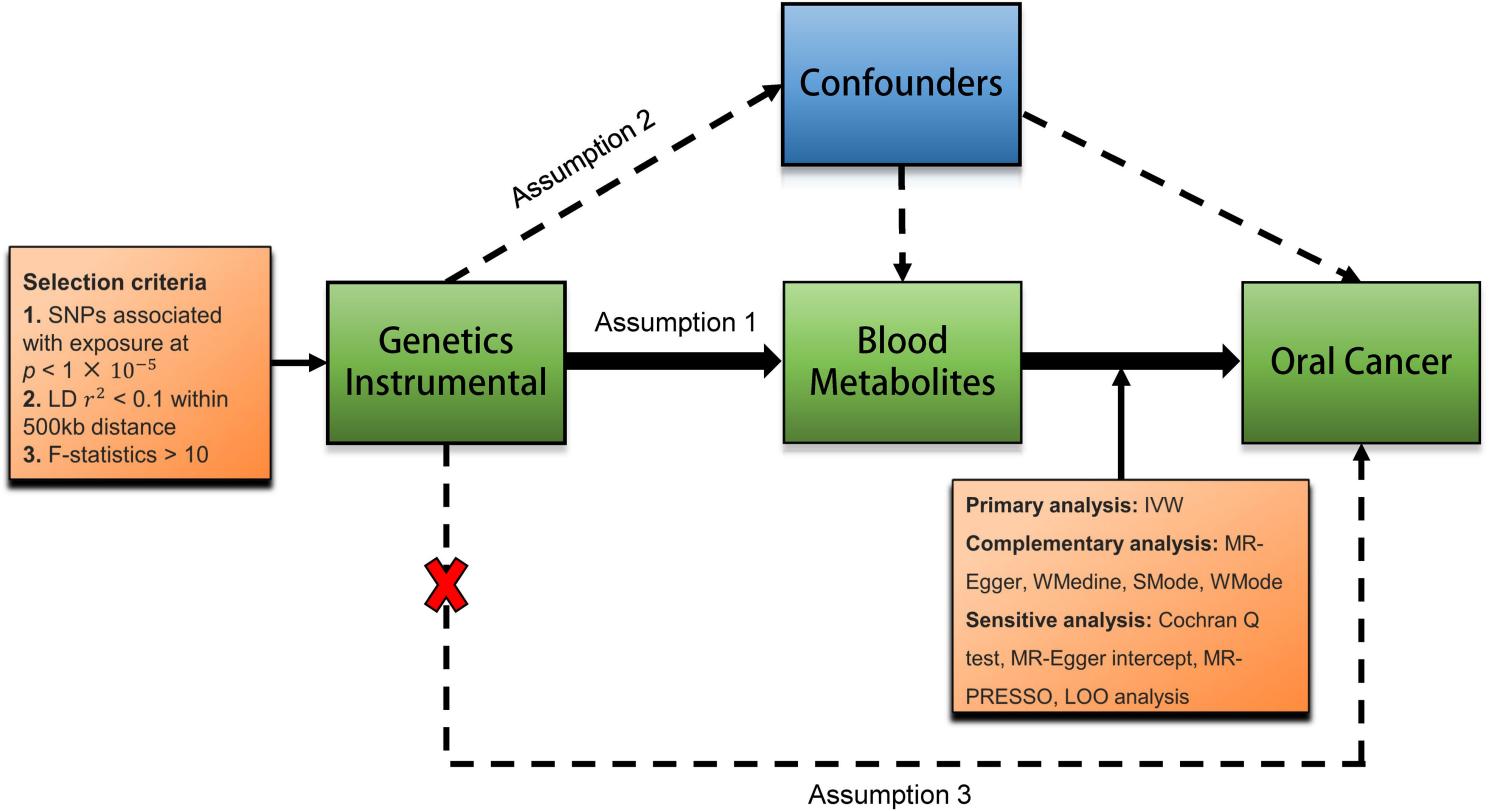
Diagram of the MR analysis. Assumption 1, genetic instruments are strongly associated with the exposures of interest; Assumption 2, genetic instruments are independent of confounding factors; Assumption 3, genetic instruments are not associated with outcome and affect outcome only via exposures. IVW, inverse variance weighted; LD, linkage disequilibrium; LOO analysis, leave-one-out analysis; MR-PRESSO, MR-Pleiotropy RESidual sum and outlier; WMedine, weighted medine; SNPs, single nucleotide polymorphisms; WM, weighted mode; SMode, simple mode.

MR study adheres to three fundamental instrumental variable (IV) assumptions: (1) the genetic variants must correlate with the exposure; (2) these variants should be free from confounding factors; and (3) they should only affect the outcome via the exposure (**Figure 1**). Data summaries for the blood metabolites and oral cancer used in this research were sourced from publicly accessible GWASs, primarily based on cohorts of European descent (8). This MR study followed the guidelines of Strengthening the Reporting of Observational Studies in Epidemiology using Mendelian Randomization (STROBE-MR).

### 2.2 Blood metabolites data synopsis

Blood metabolite genetic data were retrieved from the metabolomics GWAS server (https://metabolomics.helmholtz-muenchen.de/gwas/), which represents the most comprehensive study on blood metabolite genetic loci presently (**Supplementary Table S1**). The research by Shin et al. pinpointed around 2.1 million SNPs connected with 486 human metabolites by integrating Genome-wide association scans with high-throughput metabolic profiling (8), with metabolites prefixed by ‘X-’ having unidentified chemical properties. This research involved 7,824 Europeans: 1,768 from Germany’s KORA F4 study and 6,056 from the UK Twin Study. Among the 486 metabolites, 107 remain undefined chemically, while 309 have undergone chemical validation and are classified into eight primary metabolic groups, as cited in the Kyoto Encyclopedia of Genes and Genomes (KEGG) database. These groups encompass amino acid, carbohydrate, cofactors and vitamin, energy, lipid, nucleotide, peptide, and xenobiotic metabolism.

### 2.3 Oral cancer data synopsis

The genetic association data for oral cancer originated from the UK Biobank’s GWAS (22, 23). This initiative registered close to 500,000 participants aged between 40 and 69 (24) (**Supplementary Table S2**). The UK Biobank’s medical records utilized the International Classification of Diseases, Tenth Revision (ICD-10-CM), and Ninth Revision (ICD-9-CM). The discussed GWAS comprised a sample of 372,373 European-descent individuals, including 7.72 million SNPs. This data is accessible via the IEU GWAS database at https://gwas.mrcieu.ac.uk/. A linear mixed model (LMM) association method was applied, facilitated by BOLT-LMM (v2.3) (25). Addressing the population structure involved SNP selection post-filtration based on multiple criteria, including MAF > 0.01, genotyping rate > 0.015, a Hardy-Weinberg equilibrium p-value below 0.0001, and LD pruning to an limit of 0.1 using PLINKv2.00.

### 2.4 Selection of IVs

For the MR evaluation, SNPs linked to blood metabolites at a genome-wide significance threshold $\left(p<1\times 10^{-5}\right)$ from prior GWASs were utilized. These SNPs were chosen ensuring no linkage disequilibrium (LD) with other SNPs, maintaining an *r* ^2^ below 0.1 within a 500 kb clumping radius. In cases where SNPs exceeded the *r* ^2^ = 0.1 limit, only the SNP with the strongest association (smallest P value) with the metabolite was selected. This selection strategy is consistent with methods commonly employed in earlier studies (26, 27). To counteract potential bias from inferior instruments, the *R* ^2^ and F statistics for each SNP were determined using the following formulas:


$$R^{2}=\frac{2\times \beta^{2}\times EAF\times \left(1-EAF\right)}{\left[2\times \beta^{2}\times EAF\times \left(1-EAF\right)+2\times (se{\left(\beta^{2}\right)}^{2}\times N\times EAF\times \left(1-EAF\right)\right]}$$

$$F=\frac{N-k-1}{k}\times \frac{R^{2}}{1-R^{2}}$$

Here, β indicates the genetic variant’s effect size; EAF represents its effect allele frequency; se(β) is the standard error for this effect size; N denotes the sample size for the exposure; and k is the count of SNPs. SNPs with an F value under 10 were deemed weak and subsequently discarded. The subsequent procedure extracted outcome-associated SNPs, discarding those correlated with the outcome $\left(p<1\times 10^{-5}\right)$ . Exposure and outcome SNP harmonization followed, with the removal of palindromic and allele inconsistent SNPs. The third assumption was satisfied by excluding outcome-associated SNPs with $p<1\times 10^{-5}$ . The analysis culminated in an MR study on metabolites represented by over two SNPs (28).

### 2.5 Statistical analysis

The potential causal relationship between blood metabolites and oral cancer was explored using multiple techniques in this research. These methodologies included inverse variance weighted (IVW), MR-Egger, weighted median, weighted mode, MR-PRESSO, and simple model strategies. The overarching impact of blood metabolites on oral cancer was determined using a meta-analysis approach, combined with Wald estimates for each SNP via the IVW method. In the scenario where horizontal pleiotropy is absent, IVW results remain unbiased (29). The MR-Egger regression, based on the InSIDE assumption that instrument strength is not linked to a direct effect, gauges pleiotropy via its intercept term. An intercept term of zero in the MR-Egger regression indicates alignment with IVW results, implying an absence of horizontal pleiotropy (30). The weighted median method can provide accurate causal inferences, even if as many as 50% of the IVs are considered invalid (31). In instances where the InSIDE assumption is challenged, the weighted mode estimate offers enhanced power, reduced bias, and a lower type I error rate compared to MR-Egger regression (31). The MR-PRESSO approach identifies and corrects for horizontal pleiotropy by eliminating significant outliers. Yet, the MR-PRESSO outlier test is contingent on InSIDE premises and mandates that a majority of the genetic markers serve as valid instruments (32). While the simple mode method may exhibit lower precision, it typically presents a diminished bias relative to alternative techniques (31).

To corroborate significant outcomes, tests for heterogeneity and horizontal pleiotropy were undertaken using meta-analytical methods, including the modified Cochran Q statistic and the MR Egger intercept test for deviation (33). To alleviate the impact of horizontal pleiotropy attributed to a single SNP, a leave-one-out analysis was performed, systematically omitting one SNP at a time. The “TwoSampleMR” (34) and “MRPRESSO” R packages, version 4.1.3, facilitated these evaluations.

Furthermore, the Steiger test was executed to counteract potential biases from reverse causality (35). The causal inference direction could be erroneous if the explained variance of IVs in oral cancer surpasses that of blood metabolites.

## 3 Results

**Figure 2:**
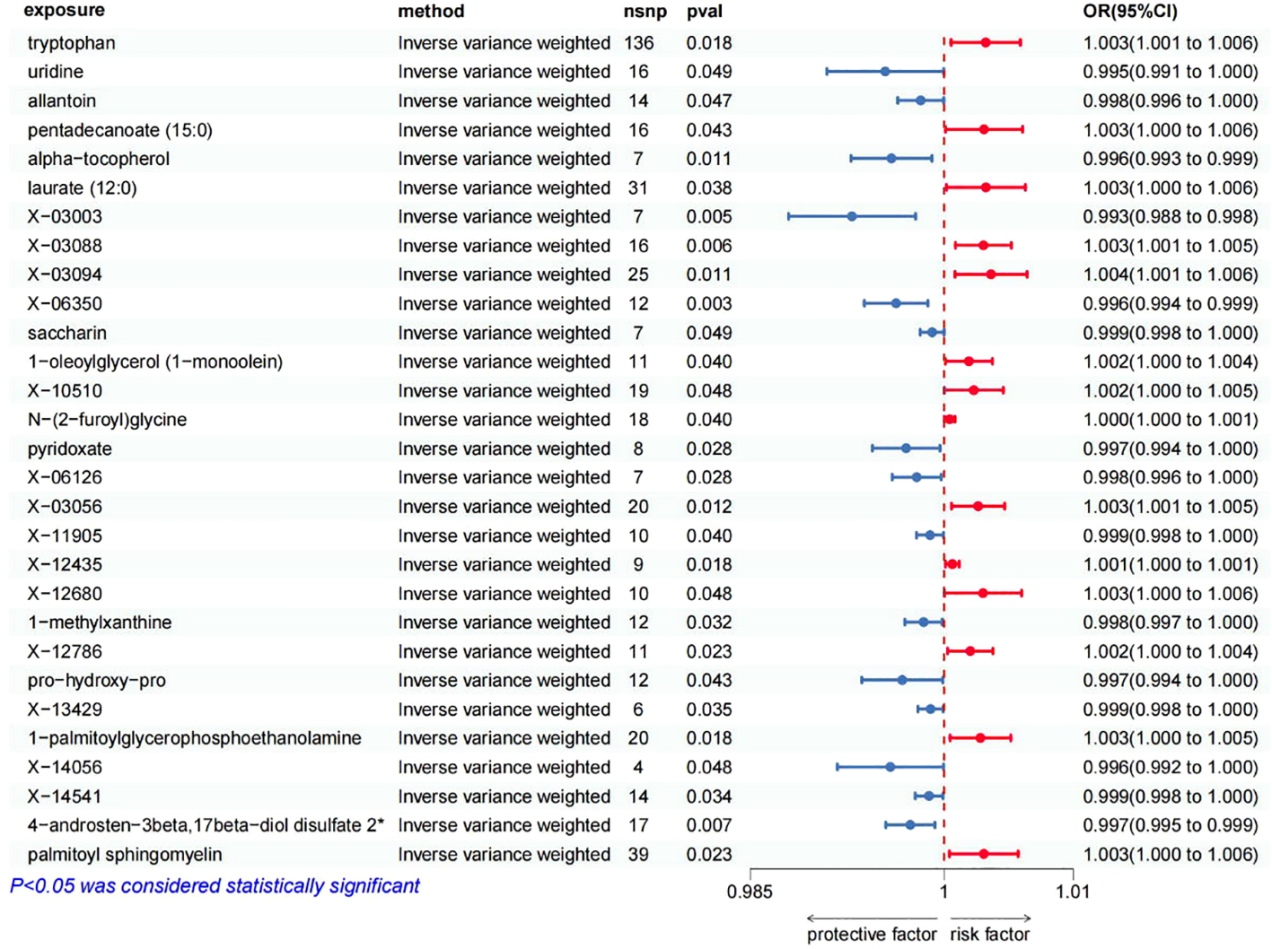
Forest plot of Mendelian randomization estimates between blood metabolites and oral cancer. The figure showed the IVW estimates of significantly oral cancer-associated blood metabolites. The blue dots represent the IVW estimates, and the blue bars represent the 95% confidence intervals of IVW estimates of protective factors. The red dots represent the IVW estimates, and the red bars represent the 95% confidence intervals of IVW estimates of risk factors.

Upon stringent quality control of IVs, 486 metabolites were included in the MR analysis. These IVs comprised SNPs ranging from 4 to 136 (with X-14056 genetically represented by 4 SNPs and tryptophan having the highest representation with 136 SNPs. The F statistics of all SNPs associated with metabolites were greater than 10 (**Supplementary Table S3**). Subsequent IVW analysis, paired with supplemental and sensitivity evaluations, pinpointed 29 metabolites that satisfied the stringent selection criteria as potential candidates (**Figure 2**). These consisted of 15 identified metabolites and 14 with unknown chemical identities. The identified metabolites were chemically categorized into Amino Acid, Vitamin, Alkaloid, Steroid, Lipid, Peptide, Nucleoside and Xenobiotics.

The positive association between the risk of oral cancer and 14 blood metabolites were as follow (**Figure 2**): tryptophan (OR 1.0032, 95% CI: 1.0005–1.0059, p=0.0183), pentadecanoate (15:0) (OR 1.0031, 95% CI: 1.0001–1.0061, p=0.0427), laurate (12:0) (OR 1.0032, 95% CI: 1.0002–1.0063, p=0.0378), X-03088 (OR 1.0030, 95% CI: 1.0009–1.0052, p=0.0059), X-03094 (OR 1.0036, 95% CI: 1.0008–1.0064, p=0.0107), 1-oleoylglycerol (1-monoolein) (OR 1.0019, 95% CI: 1.0001–1.0037, p=0.0397), X-10510 (OR 1.0023, 95% CI: 1.0000–1.0046, p=0.0479), N-(2-furoyl)glycine (OR 1.0004, 95% CI: 1.0000–1.0008, p=0.0396), X-03056 (OR 1.0026, 95% CI: 1.0006–1.0047, p=0.0121), X-12435 (OR 1.0006, 95% CI: 1.0001–1.0012, p=0.0181), X-12680 (OR 1.0030, 95% CI: 1.0000–1.0060, p=0.0481), X-12786 (OR 1.0020, 95% CI: 1.0003–1.0038, p=0.0230), 1-palmitoylglycerophosphoethanolamine (OR 1.0028, 95% CI: 1.0005–1.0052, p=0.0185), palmitoyl sphingomyelin (OR 1.0031, 95% CI: 1.0004–1.0057, p=0.0234). This suggests that these blood metabolites may increase the risk of oral cancer.

On the other hand, we found that 15 blood metabolites were associated with a reduced risk of oral cancer (**Figure 2**): uridine (OR 0.9954, 95% CI: 0.9909–1.0000, p=0.0485), allantoin (OR 0.9982, 95% CI: 0.9964–1.0000, p=0.0467), alpha-tocopherol (OR 0.9959, 95% CI: 0.9928–0.9991, p=0.0109), X-03003 (OR 0.9929, 95% CI: 0.9880–0.9978, p=0.0046), X-06350 (OR 0.9963, 95% CI: 0.9938–0.9987, p=0.0031), saccharin (OR 0.9991, 95% CI: 0.9982–1.0000, p=0.0487), pyridoxate (OR 0.9971, 95% CI: 0.9944–0.9997, p=0.0279), X-06126 (OR 0.9979, 95% CI: 0.9960–0.9998, p=0.0285), X-11905 (OR 0.9989, 95% CI: 0.9979–1.0000, p=0.0403), 1-methylxanthine (OR 0.9984, 95% CI: 0.9970–0.9999, p=0.0324), pro-hydroxy-pro (OR 0.9968, 95% CI: 0.9936–0.9999, p=0.0430), X-13429 (OR 0.9990, 95% CI: 0.9980–0.9999, p=0.0351), X-14056 (OR 0.9958, 95% CI: 0.9917–1.0000, p=0.0479), X-14541 (OR 0.9988, 95% CI: 0.9978–0.9999, p=0.0343), 4-androsten-3beta,17beta-diol disulfate 2* (OR 0.9974, 95% CI: 0.9955–0.9993, p=0.0070). This suggests that these blood metabolites may have a protective effect against oral cancer.

**Figure 3:**
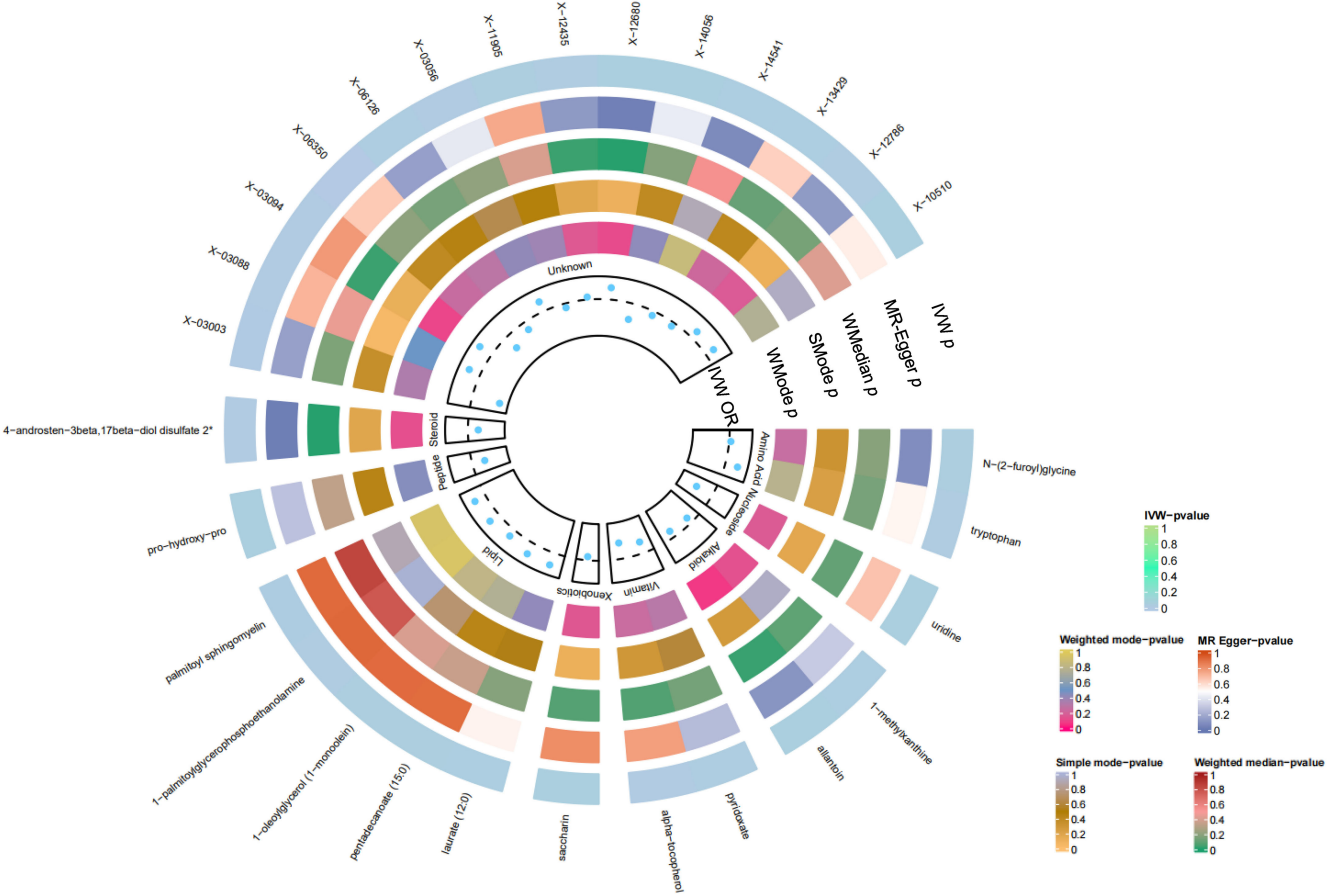
Preliminary MR analyses for the associations between blood metabolites and the risk of oral cancer. The circle from the outer to the inner represented the IVW, MR-Egger, weighted medine, simple mode and weighted mode, respectively. Blood metabolites was classified in order, Amino Acid, Nucleoside, Alkaloid, Vitamin, Xenobiotics, Lipid, Peptide, Steroid, Unknown. The shades of color were reflections of the magnitude of the p-value as the label inside the circle. (MR, Mendelian randomization; IVW, inverse variance-weighted; WMedine, weighted median; SMode, simple mode; WMode, weighted mode.).

**Figure 4:**
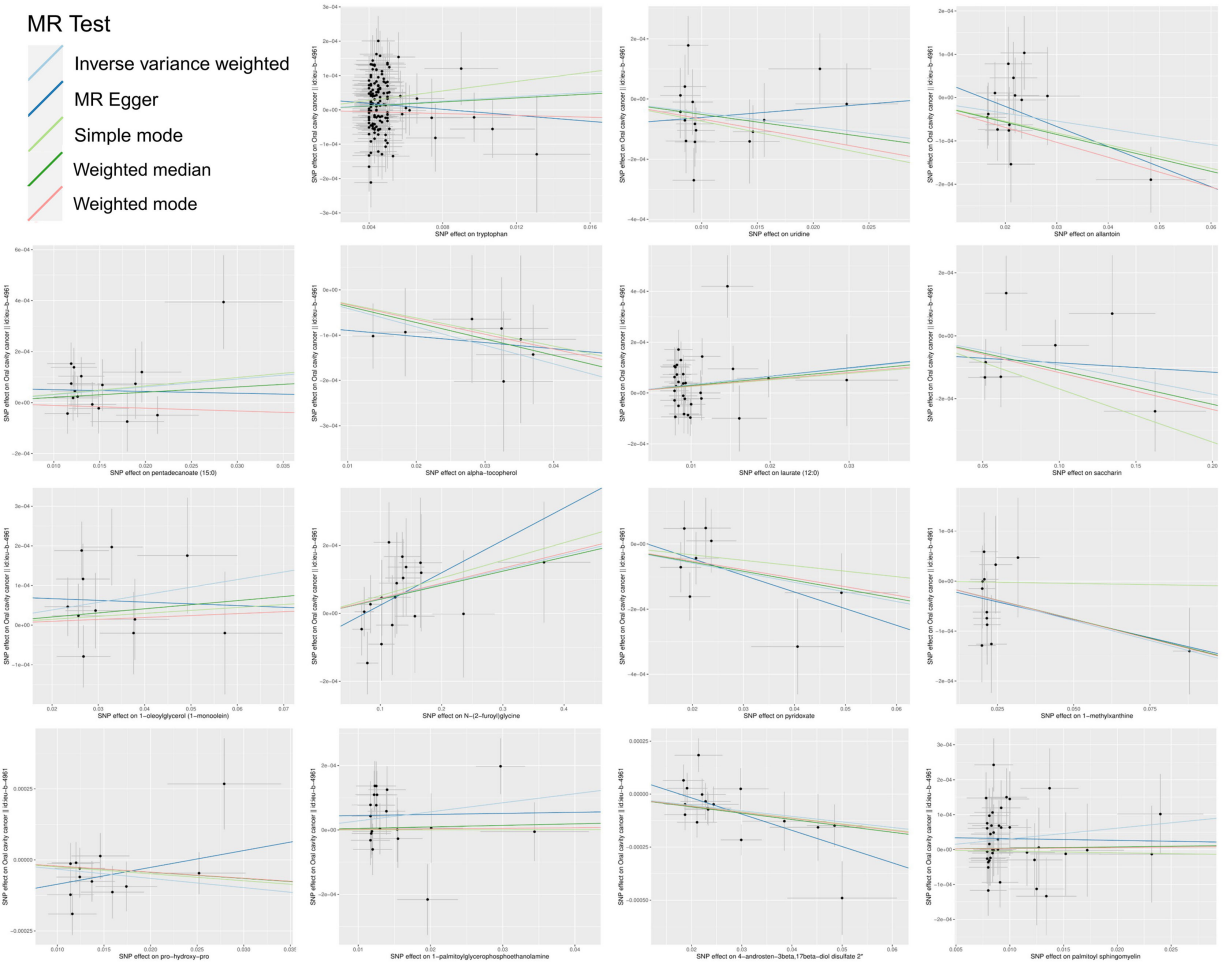
Scatter plots of the MR estimates for the significant causality of blood metabolites and the risk of oral cancer. The causal effect of the 15 identified metabolites on oral cancer. The lines implying positive correlations moved diagonally upward from left to right, indicating a facilitative effect of metabolites on oral cancer. The horizontal and vertical lines indicated each correlation’s 95% confidence interval. The lines implying negative correlations move diagonally downward from left to right, indicating the inhibitory effect of blood metabolites on oral cancer. (MR, Mendelian randomization; SNPs, single nucleotide polymorphisms).

In summation, IVW-derived estimates were significant (p<0.05), and there was consistency in direction and magnitude across IVW, MR-Egger, Weighted median, Weighted mode and Simple mode estimates (**Figure 3**; **Supplementary Table S4**). Scatter plots for identified metabolites across various tests are displayed in **Figure 4**. Both the Cochran Q test (p>0.05) and the MR-Egger intercept test (p>0.05) strongly supported the lack of heterogeneity and pleiotropy (**Table 1**). Leave-one-out analysis affirmed that no individual SNP introduced bias into the MR estimation (**Supplementary Figure S1**). The funnel plots were showed on **Supplementary Figure S2**. In addition, the Steiger test revealed that the causality between genetically proxied metabolites and oral cancer was not violated by reverse causal effects (**Supplementary Table S5**).

**Table 1:** Pleiotropy and heterogeneity of MR.

Exposure	Cochran Q	MR-Egger	MR-PRESSO
tryptophan	0.37	0.27	0.36
uridine	0.29	0.26	0.31
allantoin	0.61	0.28	0.55
pentadecanoate (15:0)	0.58	0.55	0.55
alpha-tocopherol	0.99	0.54	0.99
laurate (12:0)	0.15	0.98	0.19
saccharin	0.32	0.60	0.41
1-oleoylglycerol (1-monoolein)	0.35	0.55	0.36
N-(2-furoyl)glycine	0.79	0.28	0.81
pyridoxate	0.38	0.59	0.45
1-methylxanthine	0.84	0.89	0.89
pro-hydroxy-pro	0.32	0.09	0.39
1-palmitoylglycerophosphoethanolamine	0.36	0.45	0.38
4-androsten-3beta,17beta-diol disulfate 2*	0.09	0.10	0.09
palmitoyl sphingomyelin	0.45	0.45	0.47
X-03003	0.65	0.22	0.68
X-03056	0.31	0.58	0.37
X-03088	0.75	0.11	0.66
X-03094	0.59	0.45	0.58
X-06126	0.70	0.25	0.74
X-06350	0.41	0.21	0.48
X-10510	0.90	0.14	0.91
X-11905	0.91	0.27	0.91
X-12435	0.57	0.35	0.60
X-12680	0.07	0.08	0.09
X-12786	0.26	0.61	0.32
X-13429	0.82	0.72	0.88
X-14056	0.94	0.60	0.93
X-14541	0.28	0.18	0.30

## 4 Discussion

In recent decades, extensive research has emphasized the significant roles of metabolic reprogramming and energy metabolism in the proliferation and metastasis of oral cancer cells. Within the realm of normal cellular function, alterations in metabolism not only bolster cellular proliferation and division but also predispose cells to oncogenic transformation, a critical aspect in the context of oral cancer. In the course of conducting a Mendelian randomization study on the impact of blood metabolites on oral cancer, a multitude of blood metabolites were identified to be significantly associated with the risk of oral cancer. Our experimental results revealed that metabolites such as tryptophan, uridine, allantoin, pentadecanoate, α-tocopherol, and laurate were correlated with the risk of oral cancer.

Amino acids play a crucial role in oral cancer by providing essential building blocks and energy for tumor cell proliferation and are intimately involved in metabolic processes linked to tumor growth and survival (36). Gupta et al. discerned that metabolites including glutamine, propionate, acetone, and choline could accurately differentiate oral cancer from healthy controls, partially resonating with our findings (37). Synchronous luminescence spectroscopy has been employed to distinguish oral cancer tissues. Notable observations were made concerning peak shifts and broadening for tryptophan, NADH, and FAD in oral cancer tissues (38). These findings suggest substantial biochemical and micro-environmental alterations at the cellular level. When we pair these observations with our positive data, especially that of tryptophan (OR 1.0032, 95% CI: 1.0005–1.0059, p=0.0183), it underscores the significance of tryptophan as a crucial endogenous fluorophore in oral cancer. The changes in tryptophan not only echo the biochemical shifts within cells but might also be indicative of the progression and development of the cancer. However,. Our study also observed a down-regulation in the levels of pro-hydroxy-pro (OR 0.9968, 95% CI: 0.9936–0.9999, p=0.0430) in oral cancer patients, a finding corroborated by Yonezawa et al., who reported a down-regulation in the serum levels of several amino acids in head and neck cancer patients (39). Further analysis aligns our findings with the study by Kong et al., who through NMR analysis found that the up-regulation of lactate, choline, and glucose and the down-regulation of proline, valine, isoleucine, aspartate, and 2-hydroxybutyric acid may contribute to oral cancer development (40). This further affirms the significance of plasma metabolites as potential metabolic biomarkers for oral carcinogenesis.

Our observation is the consistent demonstration of the anticancer activity of α-tocopherol across multiple studies, especially regarding its therapeutic potential for oral cancer (41–43). α-tocopherol acts by inducing apoptosis to inhibit the growth of cancer cells. Moreover, it serves as a scavenger of free radicals, combating cancer cells through oxidative stress. While its antitumor activity may be perceived as weaker compared to cisplatin, indications of apoptosis onset appear earlier for α-tocopherol. Its lack of cytotoxicity further illustrates its potential as an antitumor agent (41). Alpha-tocopherol has displayed effective antitumor activity against ORL-48 in experiments (43). Alongside elevated serum levels of retinol, it’s been associated with a decreased risk of oral cancer. These findings suggest that alpha-tocopherol might serve as a promising therapeutic agent for oral cancer. Variations in tryptophan concentrations hint at biochemical and micro-environmental changes occurring in oral cancer tissues. In our Mendelian experiment, α-tocopherol has an OR value of 0.9959, with a 95% CI of 0.9928-0.9991 and p=0.0109, indicating a significant association with a specific condition or phenotype. This bolsters its potential therapeutic implications.

In our study, we initially linked allantoin to oral cancer with a down-regulation (OR 0.9982, 95% CI: 0.9964–1.0000, p=0.0467). While focusing on oral cancer, broader research suggests allantoin’s effects might extend to other cancers, indicating a need to understand its systemic impact beyond the oral cavity. Allantoin could increases under stress conditions, has shown cytotoxicity against various age-related cancers, especially in environments simulating oxidative stress. In our study, 1-palmitoylglycerophosphoethanolamine (OR 1.0028, 95% CI: 1.0005–1.0052, p=0.0185), has an up-regulation in oral cancer patients. In an Alpha-Tocopherol, Beta-Carotene Cancer Prevention Study, 1-palmitoylglycerophosphoethanolamine has reported a significant association with retinol levels, suggesting its potential role in the metabolic pathways influenced by vitamin A (44, 45). Randomized trials and meta-analyses indicated a potential increased risk for certain cancers with high serum retinol concentrations or after supplementation (46). Both cases highlight the importance of studying these metabolites in a wider cancer context.

Our study also unveiled results that diverge from previous research. For example, our research found a significant negative correlation between the levels of uridine and pyridoxate and the risk of oral cancer, a finding not reported in previous studies. This may suggest a diverse role of these metabolites in the onset and progression of oral cancer. Additionally, our study discovered new metabolites related to oral cancer risk, such as X-03003 and X-03088, which have not been previously reported to be associated with oral cancer risk. These novel findings may contribute to a more comprehensive understanding of the metabolic mechanisms of oral cancer and offer new potential biomarkers for the early diagnosis and prevention of oral cancer.

Several limitations exist within this study. Firstly, the exposure of interest at the genome-wide level had a restricted number of SNPs. This was addressed by applying slightly relaxed thresholds for the MR analysis, mirroring practices in previous research. Nonetheless, with F-statistic values for the chosen SNPs surpassing 10, the robustness of our IVs is indicated. The Steiger test results further affirm the validity of our threshold approach by consistently supporting the causal direction. Secondly, our MR analysis exclusively utilized GWAS data from European ancestry individuals, limiting ethnic variability. Consequently, the applicability of these findings to diverse populations necessitates further investigation and validation. Thirdly, the MR estimation’s precision is inherently tied to sample size, emphasizing the need to augment the sample size for result validation. While MR analysis sheds light on disease etiology, it’s imperative to corroborate our findings through rigorous RCTs and foundational research before clinical integration.

## 5 Conclusion

This MR study offers evidence of the role specific blood metabolites play in oral cancer, pinpointing several with potential risk or protective effects. These findings could be helpful for new diagnostic tools and treatments for oral cancer. While the results are promising, additional research is necessary to fully validate and refine these conclusions. This study serves as a foundational step towards more comprehensive understandings in the future.

## Data availability statement

The original contributions presented in the study are included in the article/**Supplementary Material**, further inquiries can be directed to the corresponding author/s.

## Ethics statement

All the data used in this research can be found in public databases. No additional ethical approval was required.

## Author contributions

ZH: Conceptualization, Formal analysis, Methodology, Project administration, Resources, Software, Supervision, Visualization, Writing – original draft, Writing – review & editing. ZX: Data curation, Investigation, Resources, Writing – original draft. QY: Data curation, Investigation, Resources, Writing – original draft. XP: Data curation, Investigation, Resources, Writing – original draft. PS: Data curation, Investigation, Resources, Writing – original draft. DZ: Data curation, Investigation, Resources, Writing – original draft. JZ: Data curation, Investigation, Resources, Writing – original draft. ZL: Conceptualization, Funding acquisition, Project administration, Writing – review & editing. RD: Conceptualization, Investigation, Project administration, Writing – original draft.
