# Supplementary figures and images for: The role of blood metabolites in oral cancer: insights from a Mendelian randomization approach
Source: Front Oncol. 2024 Feb 5;14:1305684. doi: 10.3389/fonc.2024.1305684 (PMC10876297; doi:10.3389/fonc.2024.1305684)

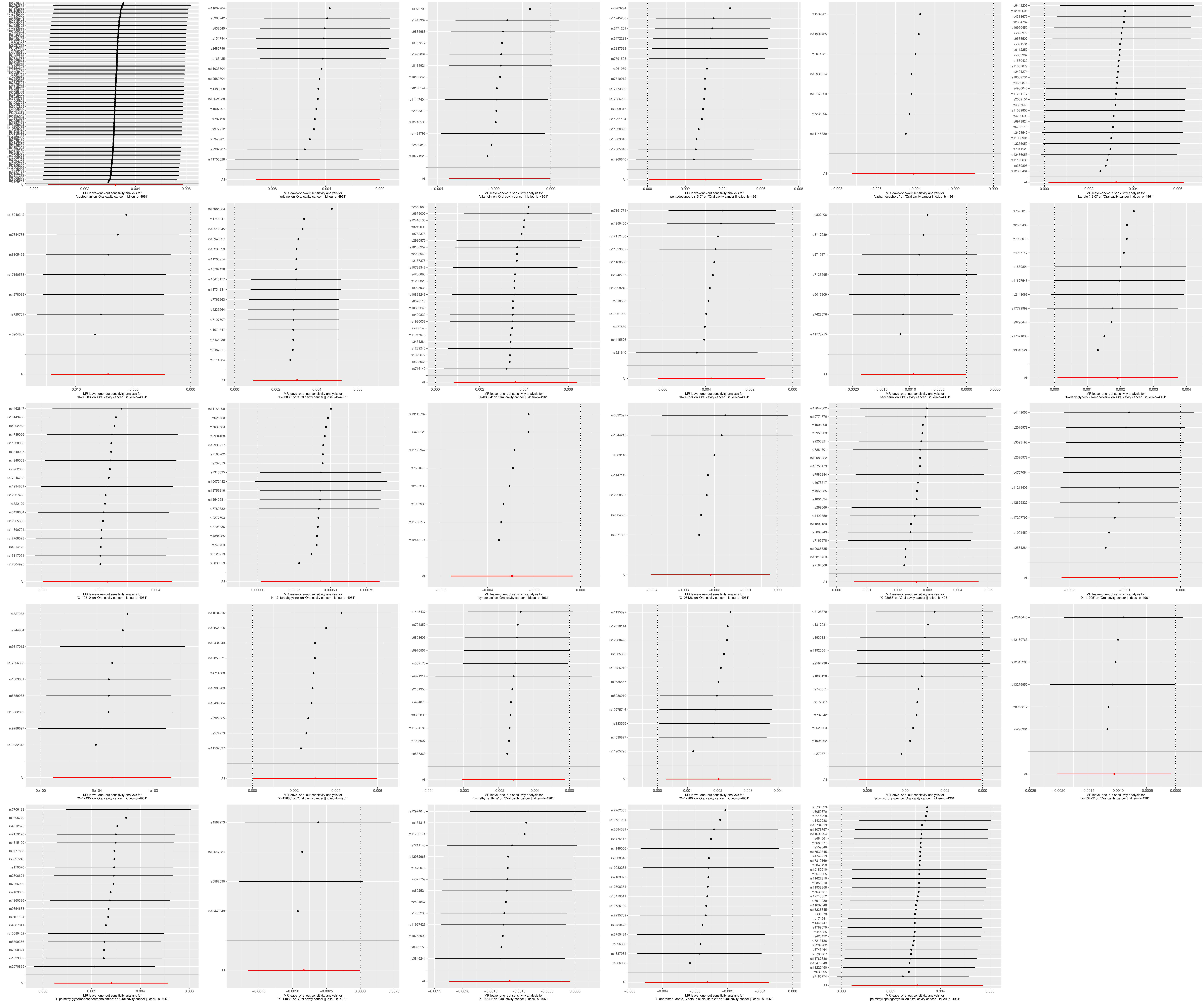

Supplement: Supplementary file 6 [file Image_1.png]
